# The Mitochondrial Permeability Transition Pore—Current Knowledge of Its Structure, Function, and Regulation, and Optimized Methods for Evaluating Its Functional State

**DOI:** 10.3390/cells12091273

**Published:** 2023-04-27

**Authors:** René Endlicher, Zdeněk Drahota, Kateřina Štefková, Zuzana Červinková, Otto Kučera

**Affiliations:** 1 Department of Physiology, Faculty of Medicine in Hradec Králové, Charles University, 500 03 Hradec Králové, Czech Republic; endlicherr@lfhk.cuni.cz (R.E.); wolff@lfhk.cuni.cz (Z.Č.); 2 Department of Anatomy, Faculty of Medicine in Hradec Králové, Charles University, 500 03 Hradec Králové, Czech Republic; stefkovka@lfhk.cuni.cz; 3 Institute of Physiology, Czech Academy of Sciences, 142 00 Prague, Czech Republic

**Keywords:** mitochondria, mitochondrial permeability transition pore, mitochondrial permeability transition, calcium signaling, calcium retention capacity, calcium-induced swelling

## Abstract

The mitochondrial permeability transition pore (MPTP) is a calcium-dependent, ion non-selective membrane pore with a wide range of functions. Although the MPTP has been studied for more than 50 years, its molecular structure remains unclear. Short-term (reversible) opening of the MPTP protects cells from oxidative damage and enables the efflux of Ca^2+^ ions from the mitochondrial matrix and cell signaling. However, long-term (irreversible) opening induces processes leading to cell death. Ca^2+^ ions, reactive oxygen species, and changes in mitochondrial membrane potential regulate pore opening. The sensitivity of the pore to Ca^2+^ ions changes as an organism ages, and MPTP opening plays a key role in the pathogenesis of many diseases. Most studies of the MPTP have focused on elucidating its molecular structure. However, understanding the mechanisms that will inhibit the MPTP may improve the treatment of diseases associated with its opening. To evaluate the functional state of the MPTP and its inhibitors, it is therefore necessary to use appropriate methods that provide reproducible results across laboratories. This review summarizes our current knowledge of the function and regulation of the MPTP. The latter part of the review introduces two optimized methods for evaluating the functional state of the pore under standardized conditions.

## 1. Introduction

Mitochondria, organelles located in the cytosol of most eukaryotic cells, have many important functions. They are equipped with enzyme systems that ensure the oxidation of various nutrients and store energy in the chemical “macroergic” bonds of adenosine triphosphate (ATP) molecules, which serve as a universal source of energy for all cells. The energy released during the splitting of an ATP molecule is, among other roles, necessary for transport processes across biological membranes. ATP production is determined by a series of chemical reactions and transport processes, and disruption of even one of these events can have fatal consequences, not only for cells but for an entire organism. The production of ATP can be disturbed by the action of oxygen radicals of both endogenous and exogenous origin. Mitochondria are involved in maintaining intracellular homeostasis, but they also produce a significant amount of reactive oxygen species (ROS) and are exposed to the effects of their action. Increased production of ROS, which is associated with the exhaustion of antioxidant protection, may lead to mitochondrial damage and cell death.

Mitochondria have an important role in regulating the cytosolic concentration of Ca^2+^ ions and in cell signaling. Mitochondria are not the primary organelle in which calcium is stored, but they have been shown to be very involved in the modulation of the Ca^2+^ signal in cells due to their ability to repeatedly receive and release Ca^2+^ ions according to the needs of the cell. The mitochondrial permeability transition pore (MPTP) plays an important role in the transport of Ca^2+^ ions into the cytosol from the mitochondrial matrix. Accumulation of Ca^2+^ ions in the mitochondrial matrix induces the opening of the pore and subsequent changes in the permeability of the inner mitochondrial membrane (IMM), which becomes highly permeable to solutes and low molecular weight substances (molecular weight up to 1.5 kDa). This phenomenon is known as the mitochondrial permeability transition (MPT) [1].

The MPTP is a supramolecular channel connecting the cytosolic and intramitochondrial spaces. At the juxtaposition of the IMM and outer mitochondrial membrane (OMM), a large complex of proteins may occur and subsequently form a pore under specific conditions. In recent years, research has focused on the transport of ions across biological membranes and, above all, on the function of the mitochondrial permeability transition pore. Although the molecular structure of the pore has still not been elucidated, it has been possible to determine the conditions under which the MPTP is activated and the factors that inhibit it. In this review, we discuss our current knowledge of the MPTP, possible models of the molecular structure of the MPTP, and the function and regulation of the MPTP. Finally, we focus on methods for evaluating the functional state of the pore and its sensitivity to regulatory factors.

## 2. The Mitochondrial Permeability Transition Pore—A Historical View

Mitochondrial membrane permeability was initially considered an artifact that arose in laboratory conditions during the isolation of mitochondria. However, further research showed that this phenomenon occurred as a result of the opening of the MPTP. The existence of this pore was first suggested in 1953, when mitochondrial swelling was observed due to various conditions during the isolation of mitochondria [2], accompanied by an increase in the permeability of the IMM and an inability to phosphorylate ADP [3]. A solid description of the pore was presented by Haworth and Hunter in their extensive work [4,5,6], along with the following conclusions: (1) the process of calcium-induced permeability of the IMM, called the mitochondrial permeability transition, leads to mitochondrial swelling; (2) the permeability of the IMM, determined by short-circuiting the opening of a hydrophilic protein pore, was named the mitochondrial permeability transition pore; (3) the pore is permeable to solutes and molecules up to 1.5 kDa; (4) activation of the MPT requires Ca^2+^ ions, which bind to the IMM from the matrix side; (5) Ca^2+^ ions compete with Mg^2+^ ions for the same binding sites; (6) Ca^2+^ ions inhibit the oxidation of NADH; and (7) the MPTP opening is partially reversible and can be inhibited by the addition of chelating agents.

The MPTP is currently defined as a multiprotein complex of the inner and outer mitochondrial membrane that forms a membrane voltage-dependent ion nonselective pore (channel). The molecular structure of the MPTP has been investigated extensively over the past 50 years, and various models of the open and closed phases have been proposed. However, genetic experiments have excluded most proteins that were thought to form the molecular basis of the pore, and these proteins are now considered to be regulatory components. Only cyclophilin D (Cyp-D) has been shown to be a crucial component of the MPTP; however, this molecule is not a structural component of the pore.

### 2.1. The First (Protein) MPTP Model

Originally, ideas about the molecular structure of the MPTP were very conclusive. The pore was thought to consist of three basic components: a voltage-dependent anion channel (VDAC), an adenine nucleotide translocator (ANT), and Cyp-D. The VDAC is a major porin localized on the OMM, an ANT is an IMM transporter, and Cyp-D is an enzyme in the matrix of mitochondria. Early studies demonstrated that an ANT could be isolated in complexes with the outer mitochondrial membrane VDAC and translocator protein (TSPO), also known as the benzodiazepine receptor [7,8]. The mitochondrial inorganic phosphate carrier (PiC) was another proposed structural component of this MPTP model. A PiC was found to bind Cyp-D and ANT and to generate Ca^2+^-induced currents (MPT) [9,10]. Subsequently, this complex (ANT, VDAC, PiC) was associated with hexokinase II (HK), mitochondrial creatine kinase (CK), and Cyp-D, and the initial MPTP model was established. The MPTP was proposed to be a complex formed at contact sites between the inner and outer mitochondrial membrane, with the ANT and VDAC forming channels in the two membranes and TSPO, PiC, HK, CK, and Cyp-D stabilizing and regulating the complex [11]. Over several decades of research on the molecular nature of the pore, other proteins were added, and different models of the pore have been presented. However, genetic studies have shown that these proposed proteins are not necessary for the function of the MPTP, but instead participate in its regulation [12,13,14,15]. Surprisingly, a recent study indicates that an ANT is a structural component of the MPTP. Suppression of the gene expression of all three ANT isoforms, as well as the gene for Cyp-D led to complete deactivation of the MPTP and indicated that the ANT may represent an independent pore responsible for a low-conductance MPTP [16].

### 2.2. The ATP Synthase Model MPTP

The MPTP model has undergone a radical change in the last ten years. The original structural components (VDAC, PiC, and TSPO) are no longer considered essential but rather regulatory components of the pore. One of the last molecules that was associated with the structure of the MPTP was ATP synthase. During an investigation of the structural nature of the MPTP formed by ATP synthase, two MPTP models were formulated practically simultaneously. The first model suggested that the MPTP is formed at the interface of two ATP synthase units (the ATP synthase dimer) [17,18]. The second model posited that the ATP synthase c-ring was the main structural unit of the MPTP [19,20,21]. The binding of Ca^2+^ and Cyp-D to ATP synthase causes a change in the conformation of monomers and destabilization of their dimeric structure. The dimer hypothesis proposes that a pore is formed at the interface between subunit g and subunit e of two interacting monomers [22]. However, this does not correspond to the formation of ATP synthase dimers, which would form the structural essence of the MPTP. Mitochondria of young organisms have a greater number of ATP synthase dimers and reduced sensitivity to MPTP activation than mitochondria of older individuals. During the aging of an organism, ATP synthase dimers unfold, which leads to a change in the arrangement of mitochondrial cristae up to their disappearance [23], a decrease in ATP production, and an increased frequency of MPTP activation [24]. The second hypothesis proposes that the MPTP is formed by calcium-induced remodeling of ATP synthase, dissociation of F0 and F1 ATP synthase, and release of the lipid plug from the c-ring, which turns into the MPTP [19,22]. The role of ATP synthase as a structural component of the pore has been the subject of a number of conflicting studies in recent years and has not yet been unequivocally confirmed [25,26,27,28,29,30,31].

### 2.3. Present Insight into the Molecular Composition of the MPTP

**Figure 1:**
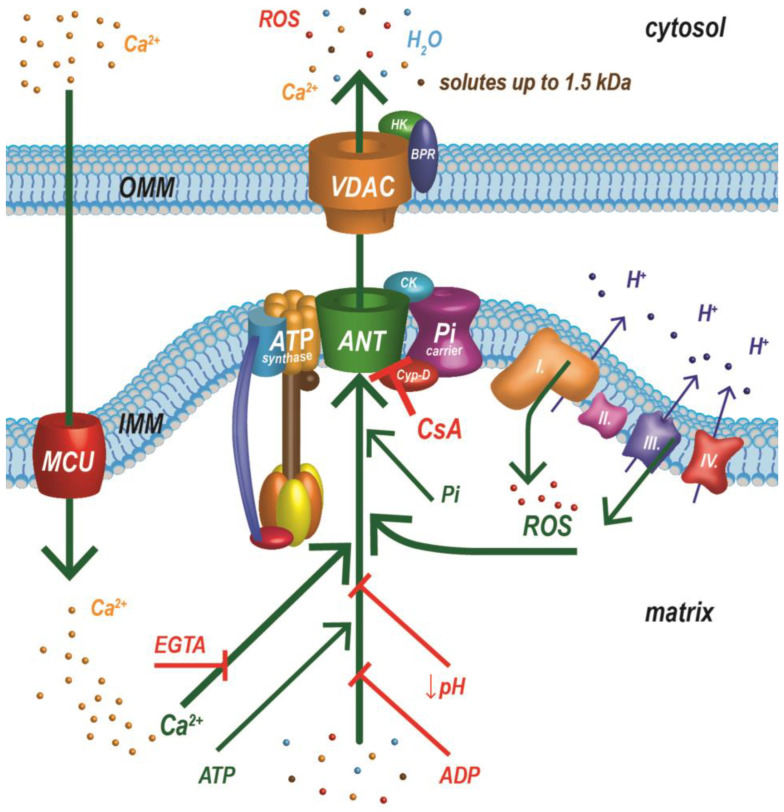
One of the latest structural models of the MPTP (ATP synthasome) and important factors regulating its functional state. The MPTP is a supramolecular complex composed of ATP synthase, a phosphate carrier, and an adenine nucleotide translocator, which is in close contact with the respiratory chain. ADP—adenosine diphosphate; ANT—adenine nucleotide translocator; ATP—adenosine triphosphate; BPR—benzodiazepine receptor (translocator protein); CsA—cyclosporin A; Cyp-D—cyclophilin D; EGTA—ethylene glycol tetraacetic acid; HK—hexokinase II; IMM—inner mitochondrial membrane; MCU—mitochondrial calcium uniporter; OMM—outer mitochondrial membrane; Pi—inorganic phosphate; PiC—inorganic phosphate carrier; Roman numerals I, II, III, and IV represent individual respiratory chain complexes; ROS—reactive oxygen species; VDAC—voltage-dependent anion channel. Green arrows indicate factors that activate MPTP opening. Red markers are for agents inhibiting MPTP opening. Modified from [34,38,39].

The different properties of the MPT support the existence of more than one group of proteins responsible for the formation of the MPTP [32]. Since we must consider an ANT again as a possible structural component of the MPTP [16], and in view of findings that mitochondria with a deficiency in the c-subunit of ATP synthase have a small channel sensitive to cyclosporine A [30], it is possible that an ANT and ATP synthase form two different MPTPs. An ANT is now thought to generate low-conductance pores [16,30], while high-conductance currents are attributed to ATP synthase pores [31]. However, we must consider the fact that an ANT and ATP synthase, along with PiC, form a large complex (ATP synthasome), and these two types of pores are closely related (Figure 1). The ATP synthasome was also proposed as a probable model of the MPTP [32,33,34,35,36,37]. The structure of the ATP synthasome adapts dynamically to the energy requirements of the cell, and recent work has shown that Cyp-D plays an important role in the dynamics of this complex. Cyp-D interacts with the three main components of the ATP synthasome, destabilizing its structure and favoring the dimeric and tetrameric forms of ATP synthase, which are less efficient for energy production. Inhibition or deletion of Cyp-D increases synthasome stability in the form of large oligomeric complexes and is accompanied by higher energy production [32]. Almost 50 years of painstaking research has not clarified the molecular nature of the MPTP. We may have to settle for the suggestions above that the MPTP does not have its own molecular basis; several different pores may be composed of different components.

## 3. MPTP Function and Properties

**Figure 2:**
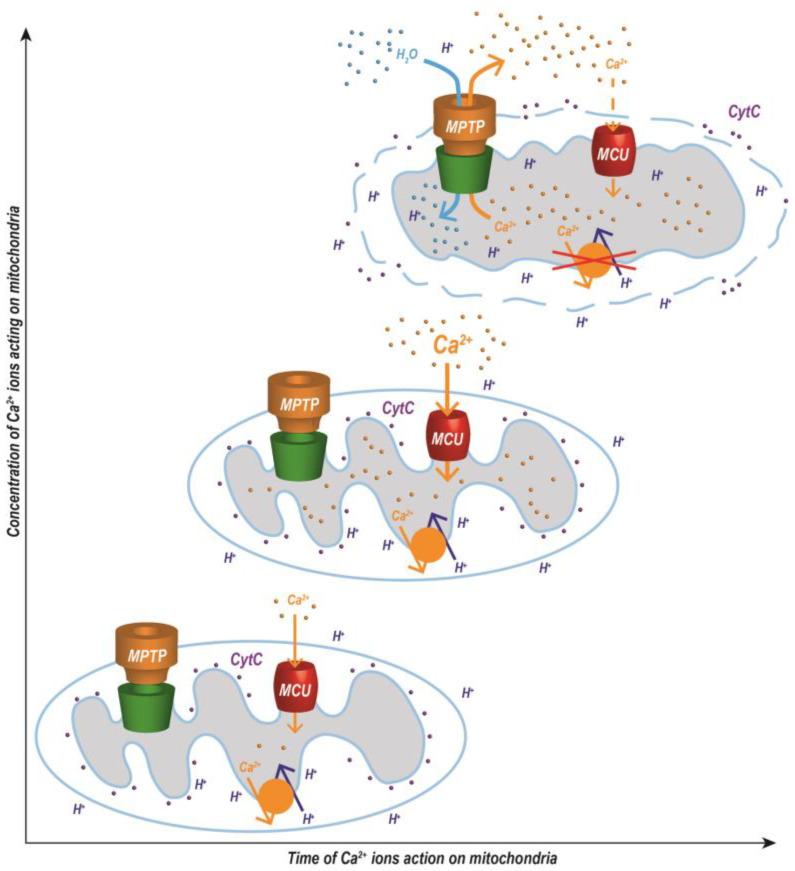
Changes in the arrangement of mitochondrial membranes induced by the opening of a Ca^2+^-dependent MPTP with subsequent osmotic processes. Mitochondrial swelling is dependent not only on the concentration of Ca^2+^ ions, but also on the time during which calcium ions act on the mitochondria. The abbreviations are defined in Figure 1. Modified from [51,52].

The MPTP is involved in the regulation of physiological and pathological cellular processes [40]. The physiological function of the MPTP has not yet been completely elucidated. The pore is involved in the regulation of Ca^2+^ ion concentration, transmembrane mitochondrial potential, and the distribution of solutes and other substances between the mitochondrial matrix and the cytosol. Short-term (reversible) opening of the pore enables the transport of protons, Ca^2+^ ions, mitochondrial ROS (mtROS), and other signaling molecules from the mitochondria to the cytosol [41]. Pore opening also protects the mitochondria from oxidative damage. A decrease in mitochondrial membrane potential (MMP) activates the respiratory chain with a subsequent decrease in the production of mtROS [42]. Long-term (irreversible) opening inhibits ATP production and induces proapoptotic and necrotic processes [43,44]. Mitochondria produce higher amounts of mtROS and even hydrolyze ATP to maintain the MMP by pumping protons into the intermembrane space via ATP synthase [45]. Complete opening of the MPTP leads to the permeability of the IMM to protons and to collapse of MMP with subsequent uncoupling of oxidative phosphorylation and cell death [46,47]. MPTP opening induces osmotic changes and massive swelling of the mitochondria, remodeling of the mitochondrial cristae, and activation of some proteins in the OMM (Bax and Bad), which cause fenestration, permeabilization, and, finally, rupture of the OMM (Figure 2). Damage to the OMM releases proapoptotic proteins from the intermembrane space into the cytosol (e.g., cytochrome c), which ultimately leads to cell death [48]. If the cell has enough ATP, these substances subsequently activate caspases, and apoptosis is induced. If the cell lacks ATP, necrosis develops [49,50].

The MPTP is regulated by voltage, and the opening of the pore is an important mechanism that affects ion and energy homeostasis of the cell. Depending on the voltage, the pore exhibits different conductance states and opens with different permeability [53]. The conductance of this channel ranges from 0.3 to 1.5 nS, with low anion selectivity; however, it can switch to a cation-selective channel [22,47,54,55]. The opening of the MPTP is, to some extent, reversible, and the pore can open transiently or permanently [56]. A low-conductance current is associated with a transient opening of the MPTP, allowing the redistribution of H^+^ and Ca^2+^ ions across the IMM. The short-term, physiological opening and closing of the pore with low permeability is referred to as a flickering MPTP and is associated with the release of signaling molecules (Ca^2+^ ions and mtROS) into the cytosol [56,57]. This phenomenon is also associated with a short-term depolarization of MMP that has consequences for the respiratory chain—activation of the respiratory chain, acceleration of the flow of electrons, and as a result, a decrease in the formation of mtROS [58]. A high-conductance current is associated with permanent opening of the pore, causing complete and permanent mitochondrial depolarization and redistribution of ions and solutes across the IMM. Damage to the supracomplexes of the respiratory chain occurs [59], which leads to a deterioration in the flow of electrons, especially through complex I, and results in increased production of mtROS [60].

Some authors confuse the terms and call these modes the low (transient) conductivity state and the high (permanent) conductivity state. However, the terms mentioned above cannot be completely confused. Transient/permanent MPTP opening is dependent on current conditions. The period of pore opening is influenced by the concentration of regulatory factors (Ca^2+^, ROS) and the time in which they act. The low/high conductivity (permeability) of the pore is determined by voltage and by the new structural arrangement of the pore, as discussed above [16,30,31]. A number of experiments have verified that the MPT phenomenon can occur differently under different conditions, supporting the hypothesis that the MPTP forms more than one molecular arrangement, each exhibiting a different conductance in the IMM. Calcium-dependent and CsA-sensitive MPT with different conductivity states has been demonstrated for each predicted MPTP arrangement. It is plausible that MPT is induced by two different types and sizes of MPTPs, through which two kinds of current pass. One pore is at low conductance, with an amplitude of 0.3 to 0.7 nS, which allows the redistribution of ions, and the second pore is at high conductance, with an amplitude of approximately 1.5 nS, which permits the passage of larger solutes (e.g., sucrose) and finally leads to cell death [22]. From these conclusions, it can be assumed that there are more MPTPs with different molecular structures.

In addition to the previously mentioned functions, the MPTP plays a key role in the aging process of organisms. There are several age-induced stimuli that activate its opening. Most often, the mitochondrial calcium and/or ROS concentration increases the frequency of MPTP opening during aging [24,61]. Oxidative stress and relatively high concentrations of Ca^2+^ ions change IMM permeability. This change in throughput leads not only to the collapse of MMP and ATP depletion, but also affects calcium signaling, as previously mentioned. It has been shown that, during aging, there are disturbances in calcium homeostasis and changes in the sensitivity of the MPTP to Ca^2+^ ions and other factors that regulate opening [62,63,64]. The increasing sensitivity of the MPTP to factors inducing its opening during the aging of an organism thus supports the hypothesis of the gradual activation of MPTP and increased cell sensitivity to proapoptotic stimuli in different organs [65,66].

## 4. Factors Regulating MPTP Opening

Accumulation of Ca^2+^ ions in the mitochondrial matrix induces pore opening and changes the permeability of the IMM, which becomes highly permeable to solutes and low molecular weight substances (molecular weight up to 1.5 kDa). MPTP opening can be reversed and is regulated by a balance between the activating and inhibitory factors. The necessary concentration of these modulators varies with different cellular conditions, setting a threshold at which MPTP opening becomes irreversible.

The functional state of MPTP is influenced by a considerable number of regulatory factors and proteins (Figure 1), as well as by post-translational modifications of these proteins. Some of them activate (open) MPTP while others inhibit it. Pore regulators are described in detail in many review articles [32,67]. Age-induced changes in the sensitivity of the pore to its regulatory factors are also significant and are clearly described in the reviews [61,63]. In the following text, we will therefore focus only on the most important regulatory factors of MPTP.

Endogenous and exogenous factors have been shown to modulate MPTP opening. The main regulatory factors include Ca^2+^ ions, ROS, and MMP, as well as inorganic phosphate and adenine nucleotides. The most important regulatory protein is Cyp-D, which activates MPTP opening. Other described regulatory proteins include mitochondrial sirtuins, creatine kinase, hexokinase II, and members of the Bcl-2 family. The strongest known inhibitors of the MPTP are cyclosporin A (exogenous) and negative mitochondrial membrane potential at physiological conditions (endogenous); other inhibitors of the pore include Mg^2+^ ions and a low pH in the mitochondrial matrix. Hundreds of publications describe the effects of individual regulatory factors on the MPTP; therefore, in our original research, we have focused on evaluating the interactions between the most important factors affecting the functional state of this pore [68,69].

Ca^2+^ ions are the most potent inducers of MPTP opening and were one of the first regulatory factors described [4,5,6]. A high concentration of Ca^2+^ ions is the primary activator of MPTP opening. However, the concentration of calcium required to open this pore is strongly dependent on other factors. The sensitivity of the MPTP to the action of calcium ions is significantly potentiated by increased oxidative stress, high concentrations of inorganic phosphate, thyroid hormones, low MMP (mitochondrial depolarization), and some other factors [69,70,71]. Although mitochondrial Ca^2+^ ions are the trigger for pore opening, under physiological conditions, mitochondria can accumulate large amounts of Ca^2+^ ions without activation of MPT. However, various endogenous modulators of the MPTP can modify the threshold concentration of Ca^2+^ ions required for opening and induce MPT even with physiological concentrations of Ca^2+^ ions [22].

As mentioned above, ROS are strong activators of MPT. Mitochondria produce not only ATP, but also a significant amount of mtROS and are exposed to their effects. mtROS may damage the complexes of the respiratory chain, causing a decrease in mitochondrial potential and ATP production. Oxidants increase the ability of Cyp-D to bind to ANT and thereby increase the sensitivity of the MPTP to Ca^2+^ ions [72,73]. The potentiating effect of ROS on calcium-induced pore opening was the subject of several of our previous original studies. Changes in calcium homeostasis and increased ROS production, associated with the exhaustion of antioxidant capacity, induce MPT and the development of diseases related to the opening of the pore. A list of diseases in which MPTP opening is involved in pathogenesis is described in detail in our overview work [74].

The relationship between calcium ions, ROS formation, and MPT induction is a complex process. As already discussed, Ca^2+^ ions induce MPTP opening, but at the same time, mitochondrial calcium overload can lead to increased production of mtROS, which increase the sensitivity of MPTP to the effects of Ca^2+^ ions. The effect of calcium overload is mostly related to the activation or inhibition of the respiratory chain, which leads to increased or decreased electron flow and electron leakage from the electron transport chain, particularly complex I and complex III [75]. Electron leakage results in the production of superoxide and other mtROS [76]. An increased supply of NADH, the complex I substrate, by calcium-stimulated activity of Krebs cycle enzymes (pyruvate dehydrogenase, isocitrate dehydrogenase, and α-ketoglutarate dehydrogenase) may also contribute to the increased activity of the respiratory chain [77]. Ca^2+^ ions have also been shown to compete with cytochrome c for binding sites on cardiolipin, which can block the electron flow from complex III to complex IV, leading to further production of mtROS [78]. Calcium and mtROS can also activate other mitochondrial enzymes (e.g., phospholipases, proteases, nitric oxide synthase, cyclooxygenase), which further contribute to increased generation of mtROS [79,80,81,82].

Cyp-D is the only proven essential regulatory component of the MPTP. Cyp-D is a soluble protein in the mitochondrial matrix and is crucial for the induction of MPTP opening [83]. To activate pore opening by Ca^2+^ ions, Cyp-D binds to components that most likely form the structural components of the MPTP (ANT, PiC, ATP synthase) [10,73,84,85]. This bond significantly reduces the threshold value of the intracellular calcium concentration, which is necessary to activate MPTP opening. Genetic studies have shown that the role of Cyp-D is essential, and opening the MPTP without this interaction requires a much higher Ca^2+^ concentration [86]. Increased expression of Cyp-D increases the pore sensitivity to Ca^2+^ ions [87], and knockout of the gene inhibits MPTP opening (reduces sensitivity to Ca^2+^ ions, PiC, and ROS) [88,89]. Cyp-D activity can be inhibited by CsA [90], but not absolutely, and MPTP opening can be activated by a high Ca^2+^ ion concentration [91].

Sangliferin A [92] and propofol [93,94] are other MPTP-inhibiting substances that are widely tested in MPT experiments. Targeted pharmacological inhibition of the MPTP could be beneficial in the treatment of diseases in which the opening of the pore is involved and could also slow aging [95]. The therapeutic use of MPTP inhibition would be useful in clinical practice, e.g., to mitigate myocardial damage due to cardiac ischemia. Since CsA must be administered before myocardial reperfusion [96], the pharmacological use of CsA in cardiovascular disease treatment is limited because the onset of acute myocardial infarction cannot be predicted in advance. However, CsA could be administered preventatively in planned cardiac surgery procedures [97]. Although a few other natural Cyp-D inhibitors have been discovered, their use has not expanded into clinical practice. Due to their toxicity, selectivity, and inappropriate pharmacokinetics, these inhibitors have not achieved the expected therapeutic results in preclinical and clinical studies. Research on suitable MPTP inhibitors has therefore focused on the development of new synthetic substances. The structure, activity, and selectivity of these new Cyp-D inhibitors are discussed in detail in a recent publication by Halečková et al. [98].

Among the endogenous factors that affect MPTP, it is worth mentioning changes in mitochondrial lipid environment [99,100], MMP [5], and the respiratory chain system. Lipid composition and organization of IMM may affect the MPTP functional status. Cardiolipin, an important phospholipid of IMM, interacts with proteins involved in MPTP formation (e.g., ANT, CypD, ATP synthase), thus influencing the properties of this pore [101,102,103]. Moreover, cardiolipin plays a crucial role in stabilizing the mitochondrial respiratory complexes [104]. Other lipids, such as cholesterol, sphingolipids, other phospholipids, and fatty acids, may also influence MPT. For example, high cholesterol levels increase the threshold for MPTP opening, while depletion of cholesterol sensitizes mitochondria to MPT induction [105]. Another example of modulators of MPTP are fatty acids, particularly long-chain polyunsaturated fatty acids [106]. It is assumed that under certain conditions, fatty acids can form the Ca^2+^-dependent but CsA-independent lipid type of MPTP in the IMM [107,108]. It is not only lipid composition and organization, but also oxidation status (e.g., lipid peroxidation) that can impact the susceptibility of MPTP to opening [109,110]. Negative MMP in physiological conditions generated by the respiratory chain ensures a supply of energy for the proper function of not only mitochondria, but the entire cell and lowers susceptibility to the activation of cell death. Intact calcium homeostasis, negative MMP, and low ROS production combined with sufficient antioxidant capacity could thus guarantee higher cell viability and perhaps even slow an organism’s aging process.

## 5. Methods for Evaluating the Functional State of the MPTP

The consequence of MPTP opening is massive mitochondrial swelling caused by osmotic changes and the flux of water from the cytosol into the mitochondrial matrix, redistribution of H^+^, and the release of accumulated Ca^2+^ ions from the mitochondrial matrix into the cytosol. This is followed by remodeling of mitochondrial cristae and eventual rupture of the OMM (Figure 2). The functional state of MPTP and its sensitivity to regulatory factors can be evaluated at the level of isolated mitochondria, living cells, and tissues. However, various methods are most often used on isolated mitochondria. These include mitochondrial swelling, mitochondrial calcium retention capacity (CRC), the patch-clamp method, evaluation of MMP changes, and indirect methods (assessment of mitochondrial oxygen consumption, measurement of cytochrome c release from the intermembrane mitochondrial space). The first method is an assessment of mitochondrial swelling, which provides information on the rate of pore opening after calcium addition and on the maximum extent of mitochondrial swelling [69,70]. The second method is based on a determination of the mitochondrial CRC, which indicates the amount of calcium needed to open the MPTP [68,71]. Both methods are most frequently used and will be discussed in more detail later. The patch-clamp method involves inserting a patch-clamp electrode into the inner mitochondrial membrane to directly measure the electrical conductance changes associated with MPTP opening. This method was repeatedly used in the search for the molecular basis of MPTP [19,55,111,112]. Specific fluorescent probes (e.g., safranin O, triphenylmethylphosphonium) are used to determine MMP in isolated mitochondria. It is important to note that mitochondrial depolarization does not always mean the MPTP opening. The MPT phenomenon can also be evaluated in living cells using a calcein-AM/CoCl_2_ assay, evaluating MMP with fluorescent probes (e.g., TMRM, TMRE, JC-1) or using imaging techniques (e.g., confocal microscopy). The calcein-AM/CoCl_2_ assay involves loading mitochondria with the fluorescent dye calcein-AM, which is sequestered in the matrix of intact mitochondria. Upon MPTP opening, calcein is released from the mitochondria and quenched by cobalt ions, resulting in a decrease in the fluorescence signal [113]. In tissues, the opening of MPTP can be studied by determining the metabolism of ^3^H 2-deoxyglucose. This method is successfully used, e.g., in the monitoring of ischemia-reperfusion damage to the heart tissue [114]. All the methods used for monitoring MPTP opening have limitations and require careful interpretation of the results. Therefore, it is recommended to use multiple methods to confirm the occurrence of MPTP opening.

### 5.1. Optimization of the Mitochondrial Swelling Method

**Figure 3:**
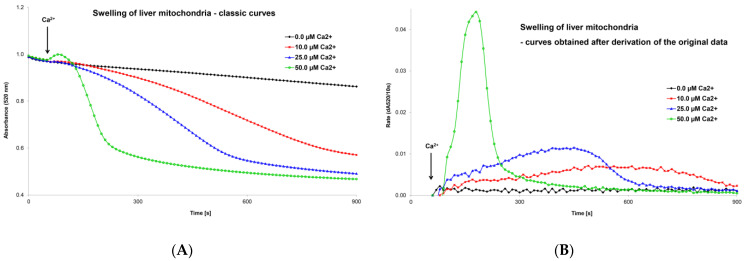
Mitochondrial swelling induced by calcium ions—classic curves (**A**) and curves obtained after derivation of primary data (**B**). The recordings show the effect of Ca^2+^ ion concentration on the extent and rate of changes in mitochondrial swelling. Isolated liver mitochondria (0.2 mg protein/mL) were incubated in swelling medium (125 mM sucrose, 65 mM KCl, and 10 mM HEPES at pH 7.2) supplemented with 0.1 mM inorganic phosphate and 10 mM succinate. Swelling was induced by adding calcium chloride between 50–60 s to the final concentration shown in the figure. Mitochondrial swelling was estimated from a decrease in the absorbance of the mitochondrial suspension at 520 nm using a Shimadzu UV 160 spectrophotometer. From these records, it is possible to obtain precise numerical values for a detailed analysis of the effect of MPTP regulatory factors. An evaluation of the extent and rate is discussed in detail in the text and in our original publications [69,70].

Very soon after the discovery of the MPT phenomenon, the kinetics of MPTP opening began to be assessed spectrophotometrically (at 520 nm) as the swelling of isolated mitochondria. A turbidimetric method was used to evaluate a decrease in absorbance of the mitochondrial suspension after the addition of calcium chloride [115,116]. The decrease in absorbance over time (the graphic curve) was the output of the measurement, and the swelling process was assessed based on changes in the shape of this curve (Figure 3A). Classic swelling curves provided qualitative information but could not provide precise quantitative data on the rate of the swelling process. This graphical record was sufficient to evaluate the various factors that activate or inhibit MPT but was insufficient to compare the simultaneous impact of more regulatory factors or their interactions on MPTP opening. In an original article, we presented an optimized mitochondrial swelling method [70]. Evaluation of mitochondrial swelling may be improved by taking the derivative of a classical swelling curve. The maximum extent of mitochondrial swelling can be obtained (in numerical form) directly from a classical swelling curve as the final change in absorbance over the following period (dA520/time). After deriving the primary data, we obtained a new modified swelling curve (Figure 3B). Two other parameters can be determined numerically from this graphical record: the maximum swelling rate, expressed as the change in absorbance per unit of time (i.e., dA520/10 s), and the time at which the swelling rate reaches its peak value after the addition of an MPT inducer or inhibitor. This optimized mitochondrial swelling method is discussed in more detail in our previously published work [70]. We used this innovative methodology to evaluate the effects of ROS and triiodothyronine on calcium-induced mitochondrial swelling [69]. We also assessed tissue specificity [117], age-dependent differences [118,119], and differences between male and female tissues [120] to calcium- and ROS-induced pore opening and evaluated ischemia/reperfusion injury [121]. Mitochondrial swelling is also used in our laboratory of experimental hepatology to evaluate changes in mitochondrial metabolism after exposure to hepatotoxic and hepatoprotective substances. Specifically, we used this method to evaluate the functional state of mitochondria after phenformin administration [122].

### 5.2. Optimization and Unification of the Calcium Retention Capacity Method

**Figure 4:**
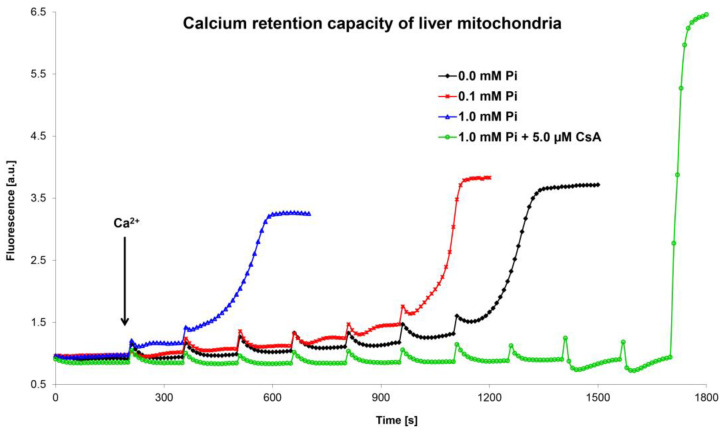
Calcium retention capacity of liver mitochondria. The figure shows representative curves of the combined effect of Ca^2+^ ions and inorganic phosphate on the mitochondrial calcium retention capacity. The mitochondrial calcium retention capacity was evaluated using the membrane-impermeable fluorescent probe calcium green-5N on an AMINCO-Bowman Series 2 spectrofluorometer (λex 506 nm, λem 592 nm). Calculation of the calcium retention capacity of isolated mitochondria was performed by multiplying the amount of added calcium chloride and the number of Ca^2+^ additions, related to mitochondrial protein concentration. Isolated liver mitochondria (0.4 mg protein/mL) were incubated in swelling medium supplemented with 10 mM succinate, 1 μM calcium green-5N, and inorganic phosphate and cyclosporine A, as shown in the figure. The amount of one Ca^2+^ addition was 1.25 nmol (in 1 mL volume). Only MPTP opening in the presence of CsA was induced by high amounts of added calcium (8 × 1.25 nmol Ca^2+^; the last two additions were 10 and 30 nmol Ca^2+^, respectively). An evaluation and calculation of calcium retention capacity is discussed in detail in the text and in our original publications [68,71].

In the last twenty years, another method was developed to assess the functional state of the MPTP. This method is based on a determination of the calcium retention capacity using Ca^2+^-sensitive dyes (e.g., calcium green-5N, arsenazo III, Oregon Green 488 BAPTA-1, Fluo-5N) or a Ca^2+^-selective electrode [41,123,124,125,126]. CRC indicates the amount of calcium ions that must accumulate in mitochondria to induce MPTP opening (Figure 4). The methods using Ca^2+^-sensitive fluorescence dyes employ the gradual addition of small amounts of calcium ions to the mitochondrial suspension in the presence of a fluorophore. Each calcium addition leads to a transient increase in fluorescence, which quickly disappears since calcium is accumulated by the mitochondria. After adding a critical (last) quantum of Ca^2+^ ions, which induces MPTP opening, the accumulated calcium from the mitochondria is released, leading to a sudden, permanent increase in fluorescence.

The CRC method based on calcium green-5N is widely used in our laboratory to evaluate the functional state of mitochondria after exposure to various substances and conditions [127]. Our CRC data on the effects of Ca^2+^ ions, inorganic phosphate, and ROS on MPT are in agreement with the results obtained during mitochondrial swelling measurements [71]. However, we found that the CRC values from rat liver mitochondria were highly dependent on the experimental conditions used. The method of measuring CRC has not yet been standardized; therefore, data obtained by various laboratories cannot be compared easily. Some laboratories prefer high peaks caused by the addition of more calcium ions, which open the pore faster [125]. Other laboratories prefer low peaks induced by lower amounts of calcium, which take longer to reach full pore opening [128].

In our two original publications, we demonstrated that CRC values from rat liver mitochondria were highly dependent on the experimental protocol used. CRC depends on the composition of the incubation medium, the amount of calcium used for titration, and the time interval between calcium additions. We also found that additional information about the process of pore opening during titration with calcium may be obtained from a detailed analysis of the fluorometric curves. By repeatedly adding quanta of Ca^2+^ ions to the mitochondria, we can assess the kinetics of MPTP opening. Our results show that there were different intermediate states of pore opening. The efflux of Ca^2+^ ions from the mitochondria was not constant, and the MPTP opened with varying intensity. In our graphic recordings, we registered two different rates of pore opening (slow and fast passage of Ca^2+^ ions through the MPTP). However, by one-shot addition of Ca^2+^ ions to the mitochondria, we can also evaluate the kinetics of MPTP opening. Our results show that not only the amount of added calcium, but also the time during which Ca^2+^ ions acted on the pore affected MPTP opening. Our data show that very low concentrations of accumulated calcium, which are thought to be ineffective during the initial opening of the MPTP, can initiate the opening of the pore if exposed for a longer period. The time required for pore opening after the accumulation of calcium in the mitochondria is dependent (shortened) on the amount of calcium added (one-time addition). It was also evident from these records that two different pore opening rates occurred for all our single CaCl_2_ additions. Our data confirm that the opening of the pore occurs in several phases, which are dependent on the amount of calcium in the mitochondrial matrix and the time in which it acts. We also hypothesize that a dose-dependent period is required before calcium accumulation in the matrix activates full pore opening [68,71].

## 6. Conclusions

The molecular structure of the MPTP has not yet been unequivocally identified. Since none of the proteins studied thus far is absolutely essential for MPTP function, it appears that there is more than one type of MPTP whose opening leads to changes in the IMM of varying degrees and duration. However, from a functional point of view, knowledge of the properties and regulating factors is more important than the exact structure of the pore. Two high-quality methods are used to study of the functional properties of the MPTP: the mitochondrial swelling method and the calcium retention capacity method. These methods can be used to evaluate organ and species specificities of the MPTP under various physiological and pathological conditions and age-induced changes in the sensitivity of the pore. The mitochondrial swelling method optimized in our laboratory allows us to quantify the obtained results. Optimization and standardization of this method is a basic prerequisite for comparing results from different laboratories and unifying conclusions on MPTP regulation. The method for determining the calcium retention capacity of mitochondria provides very accurate information about the kinetics of MPTP opening. However, it must also be standardized across laboratories to produce comparable results and avoid incorrect conclusions. Both methods are used in the search for pharmacological inhibitors of the MPTP that may be used in the treatment of diseases related to MPTP opening or in slowing an organism’s aging process.

## Data Availability

Not applicable.
